# The Receptor for Urokinase Regulates TLR2 Mediated Inflammatory Responses in Neutrophils

**DOI:** 10.1371/journal.pone.0025843

**Published:** 2011-10-05

**Authors:** Gang Liu, Yanping Yang, Shanzhong Yang, Sami Banerjee, Andressa De Freitas, Arnaud Friggeri, Kasey I. Davis, Edward Abraham

**Affiliations:** 1 Department of Medicine, University of Alabama at Birmingham, Birmingham, Alabama, United States of America; 2 Department of Pediatrics, University of Alabama at Birmingham, Birmingham, Alabama, United States of America; 3 Pole Anesthésie Réanimation, CHU d'Amiens and INSERM, ERI-12, Amiens, France; Abramson Research Center, United States of America

## Abstract

The urokinase-type plasminogen activator receptor (uPAR), a glycosylphosphatidylinositol (GPI) anchored membrane protein, regulates urokinase (uPA) protease activity, chemotaxis, cell-cell interactions, and phagocytosis of apoptotic cells. uPAR expression is increased in cytokine or bacteria activated cell populations, including macrophages and monocytes. However, it is unclear if uPAR has direct involvement in the response of inflammatory cells, such as neutrophils and macrophages, to Toll like receptor (TLR) stimulation. In this study, we found that uPAR is required for optimal neutrophil activation after TLR2, but not TLR4 stimulation. We found that the expression of TNF-α and IL-6 induced by TLR2 engagement in uPAR-/- neutrophils was less than that in uPAR+/+ (WT) neutrophils. Pretreatment of neutrophils with PI-PLC, which cleaves GPI moieties, significantly decreased TLR2 induced expression of TNF-α in WT neutrophils, but demonstrated only marginal effects on TNF-α expression in PAM treated uPAR-/- neutrophils. IκB-α degradation and NF-κB activation were not different in uPAR-/- or WT neutrophils after TLR2 stimulation. However, uPAR is required for optimal p38 MAPK activation after TLR2 engagement. Consistent with the in vitro findings that uPAR modulates TLR2 engagement induced neutrophil activation, we found that pulmonary and systemic inflammation induced by TLR2, but not TLR4 stimulation is reduced in uPAR-/- mice compared to WT counterparts. Therefore, our data suggest that neutrophil associated uPAR could be a potential target for treating acute inflammation, sepsis, and organ injury related to severe bacterial and other microbial infections in which TLR2 engagement plays a major role.

## Introduction

Host immune cells, including macrophages and neutrophils, recognize and respond to microbial pathogen invasion using pattern recognition receptors (PRRs), including Toll like receptors (TLRs), Nod-like receptors (NLRs), and RIG-I-like receptors (RLRs) [1], [2], [3], [4], [5]. TLRs were the first group of PRRs identified more than a decade ago [5]. Each TLR binds to and is activated by a unique spectrum of molecules, termed pathogen associated molecular patterns (PAMPs), that are present in microbial organisms [1], [2], [3], [4]. TLR2 and TLR4 recognize major molecular components, such as lipoglycans, lipoteichoic acid (LTA), peptidoglycan, and lipopolysaccharide (LPS), that are present on the surface of gram-positive and gram-negative bacteria [1], [2], [3], [4]. More recently, a number of intracellular molecules, such as HMGB1 and heat shock proteins, that are released to extracellular milieu by activated cell populations, have been shown to bind and activate specific TLRs [6], [7]. These endogenous molecules have been termed as damage associated molecular patterns (DAMPs) [6], [7]. Engagement of TLR2 or TLR4 leads to activation of transcription factors, including NF-κB, and enhances the production of pro-inflammatory and immunoregulatory cytokines [1], [2], [3], [4].

The urokinase-type plasminogen activator receptor (uPAR) was initially identified through its interaction with urokinase-type plasminogen activator (uPA), and consists of three globule like domains (D1, D2, and D3), but lacks trans-membrane and cytoplasmic structures [8], [9], [10]. Instead, uPAR is anchored on the cell membrane through a glycosylphosphatidylinositol (GPI) moiety [8], [9], [10]. uPAR can be released from the plasma membrane by GPI-specific phospholipase C or D to form soluble uPAR (suPAR) [8]. uPAR itself does not transduce extracellular signals. However, association between uPAR and other membrane proteins, such as integrins, enables uPAR to participate in the activation of downstream signaling events [8], [9].

A primary function of uPAR is binding to uPA and thereby concentrating the protease activity of uPA on the leading edge of migrating cells [8], [9], [10]. However, uPAR has a number of protease-independent activities [8], [9], [10]. For example, uPAR regulates cell migration, chemotaxis, cell-cell interaction, and phagocytosis of apoptotic cells through its interaction with integrins [8], [9], [10], [11], [12], [13], [14].

uPAR expression is increased in cytokine or bacteria activated cell populations including macrophages and monocytes, and contributes to the infiltration of inflammatory cells into infected tissues or organs [15], [16], [17], [18], [19]. However, it is unclear if uPAR has direct involvement in the response of inflammatory cells, such as neutrophils and macrophages, to TLR stimulation. Here, we found that uPAR is required for optimal neutrophil activation through TLR2 engagement.

## Results

### uPAR is required for neutrophil activation upon TLR2 stimulation

In our previous studies, we found that uPA and plasminogen activator inhibitor 1 (PAI-1) enhance the inflammatory response of neutrophils to TLR4 stimulation [20], [21], [22]. Furthermore, we and others demonstrated that vitronectin participates in cellular activation upon both TLR2 and TLR4 stimulation [23], [24]. Because uPA, PAI-1, and vitronectin are either ligands for, or indirectly associated with uPAR [25], we therefore examined the role of uPAR in the regulation of cellular activation after TLR2 or TLR4 engagement.

**Figure 1:**
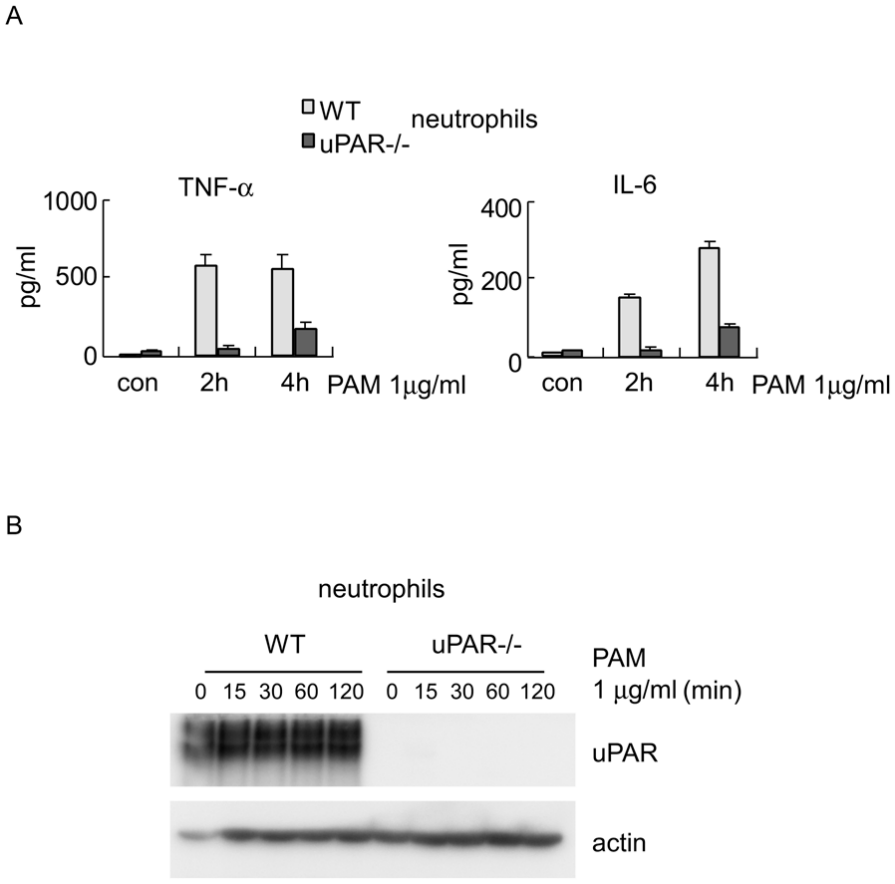
uPAR is required for neutrophil activation upon TLR2 stimulation. (A) 1×10^6^ WT and uPAR-/- neutrophils were treated with 1 µg/ml PAM for 0, 2, or 4 hours, and the levels of TNF-α and IL-6 in the supernatants determined. Neutrophils from 3–4 mice were pooled and equally divided into indicated treatment groups. n = 3 for each condition; Mean ± SD. The levels of TNF-α and IL-6 in the supernatants of stimulated uPAR-/- neutrophils were significantly less than those of stimulated WT cells. One representative experiment is shown. Three other independent experiments provided similar results. (B) 4×10^6^ WT and uPAR-/- neutrophils were treated with 1 µg/ml PAM for 0, 15, 30, 60 or 120 minutes. The cells were collected and cellular extracts prepared. The levels of uPAR were determined by Western blotting. The levels of actin were used as loading controls.

Bone marrow neutrophils isolated from wild type (WT) and uPAR knockout (uPAR-/-) mice were stimulated with the specific synthetic TLR2 ligand, PAM3CSK4 (PAM). As shown in Figure 1A, uPAR-/- neutrophils produced less TNF-α and IL-6 after PAM stimulation than did WT neutrophils. These data suggest that uPAR participates in neutrophil activation in response to TLR2 stimulation. Of note, we confirmed that uPAR-/- neutrophils showed no uPAR expression (Figure 1B).

### uPAR cleavage by PI-PLC attenuates neutrophil activation upon TLR2 stimulation

**Figure 2:**
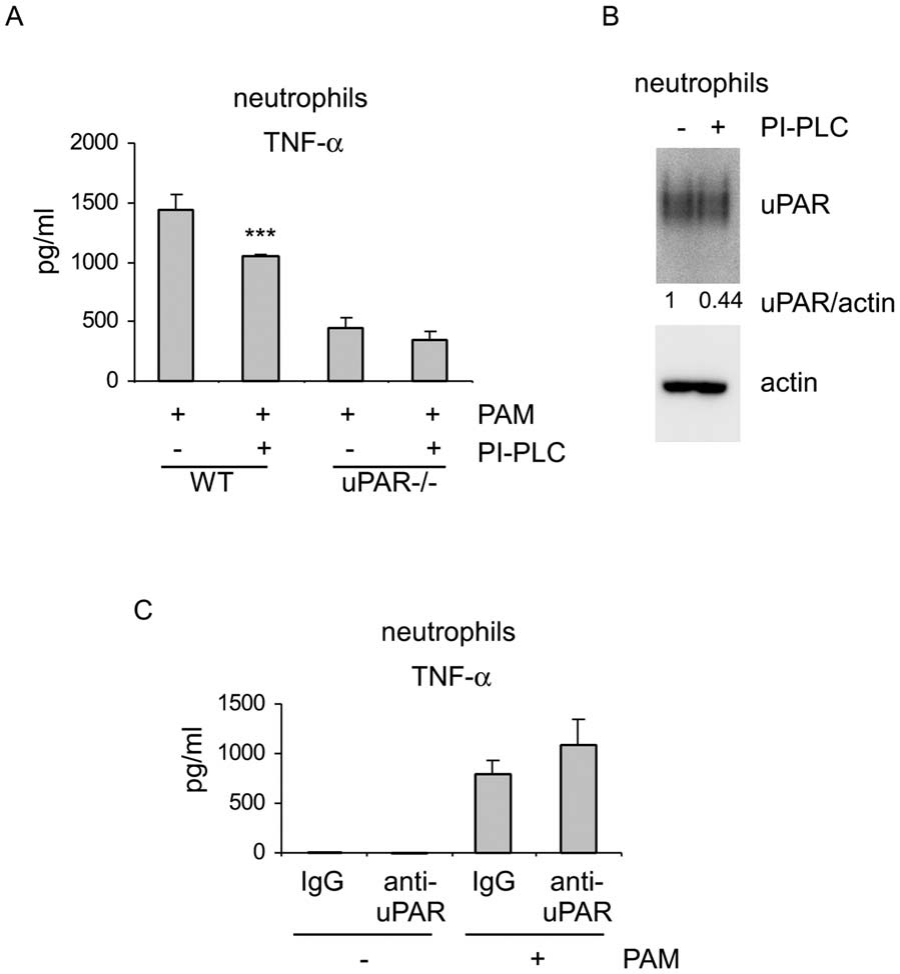
uPAR cleavage by PI-PLC attenuates neutrophil activation upon TLR2 stimulation. (A) 2×10^6^ WT and uPAR-/- neutrophils were pre-treated without or with 1 U/ml PI-PLC for 1.5 hours. The cells were then collected by centrifugation, washed twice with PBS, and treated with 1 µg/ml PAM for 4 hours. The supernatants were collected and the levels of TNF-α determined. n = 3 for each condition; Mean ± SD; ***p<0.001 when compared to WT neutrophils pretreated without PI-PLC. One representative experiment is shown. A second independent experiment provided similar results. (B) Experiments were performed as in A. Levels of uPAR and actin were determined by Western blotting. Densitometric analysis was performed, and the ratios of uPAR/actin calculated. The uPAR/actin ratio in untreated group was considered to be 1. The values in PI-PLC treated cells are shown as the ratio to that of the untreated group. Representative blots from two independent experiments are shown. (C) 2×10^6^ WT neutrophils were pre-treated with 5 µg/ml control IgG or anti-uPAR antibodies for 1 hour. The cells were then collected by centrifugation, washed twice with PBS, and treated without or with 1 µg/ml PAM for 4 hours. The supernatants were collected and the levels of TNF-α determined.

uPAR is a GPI anchored protein and can be released from the plasma membrane by the GPI-specific phospholipases C (PI-PLC) and D [8]. We reasoned that neutrophils isolated from WT mice after cleavage of uPAR with PI-PLC may resemble uPAR-/- neutrophils in their response to TLR2 stimulation. To test this hypothesis, WT neutrophils were pre-treated with PI-PLC and then stimulated with PAM. As shown in Figure 2A, PI-PLC pretreatment significantly diminished PAM stimulated TNF-α expression. However, PI-PLC pretreatment had only minimal effects on PAM induced TNF-α expression in uPAR-/- neutrophils. These data suggest that uPAR, but not other GPI anchored proteins, has a major role in neutrophil activation upon TLR2 stimulation. Of note, PI-PLC treatment decreased uPAR levels on WT neutrophils (Figure 2B).

To determine if direct interactions between uPAR and its ligands are required for the involvement of uPAR in TLR2 induced neutrophil activation, WT neutrophils were pre-treated with anti-uPAR or control antibodies and then stimulated with PAM. As shown in Figure 2C, anti-uPAR antibodies had minimal effects on PAM stimulated TNF-α expression in WT neutrophils. These data suggest that the ability of uPAR to enhance neutrophil activation by TLR2 is independent of association of uPAR with its ligands, but rather requires the structural presence of uPAR that presumably interacts in a trans fashion with other membrane associated ligands.

### uPAR is not involved in the transcription of pro-inflammatory cytokines upon TLR2 stimulation in neutrophils

**Figure 3:**
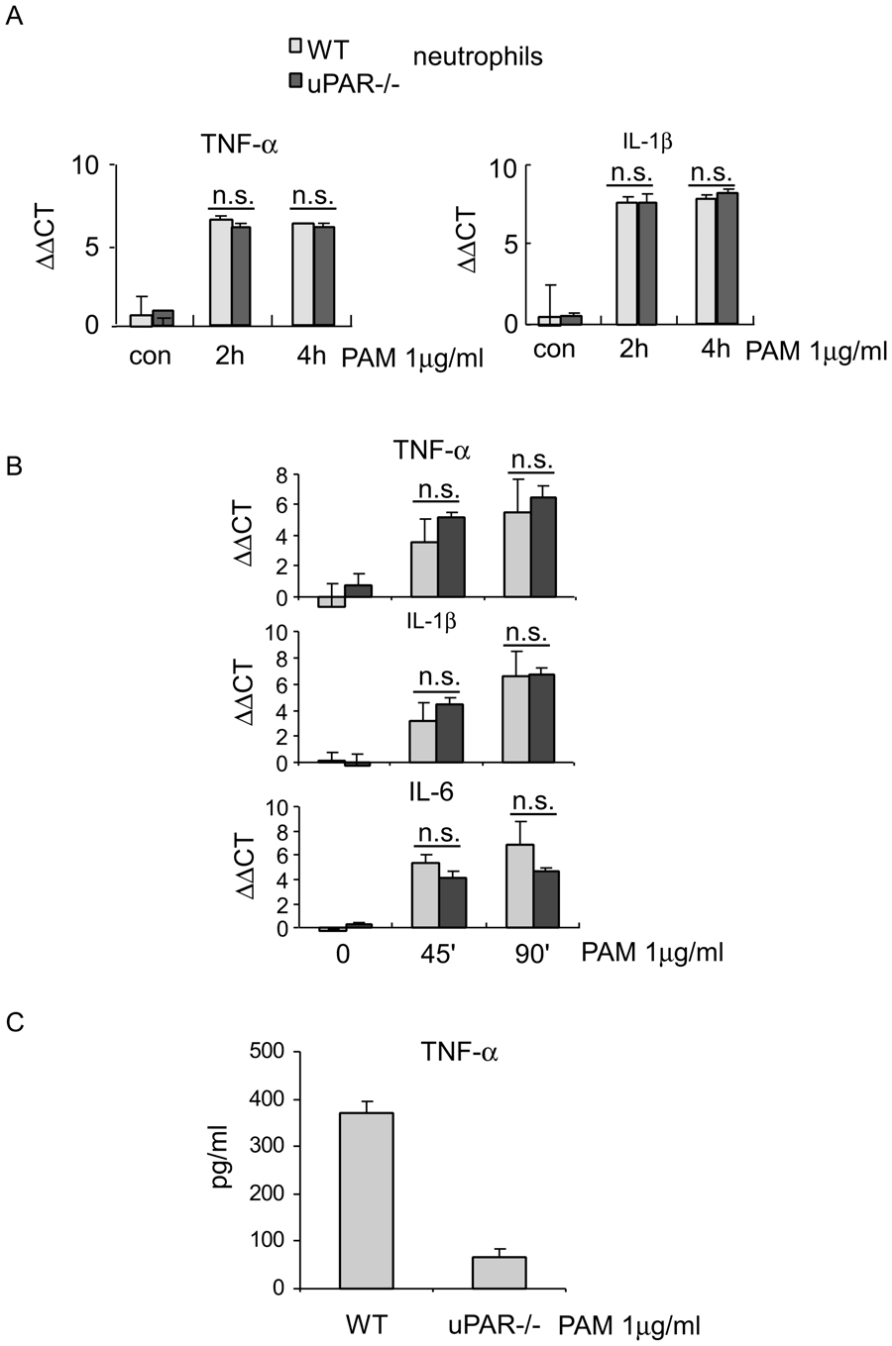
uPAR is not involved in the transcription of pro-inflammatory cytokines upon TLR2 stimulation in neutrophils. (A) 1×10^6^ WT and uPAR-/- neutrophils were treated with 1 µg/ml PAM for 0, 2, or 4 hours. The cells were collected and total RNA from the cells purified. The mRNA levels of TNF-α and IL-1β in the cells were determined by real-time PCR. n = 3 for each condition; Mean ± SD; All p values were greater than 0.05. (B) 1×10^6^ WT and uPAR-/- neutrophils were treated with 1 µg/ml PAM for 0, 45, or 90 min. The mRNA levels of TNF-α, IL-1β, and IL-6 in the cells were determined by real-time PCR. n = 3 for each condition; Mean ± SD; All p values were greater than 0.05. (C) 1×10^6^ WT and uPAR-/- neutrophils were treated with 1 µg/ml PAM for 4 hours. The supernatants and the neutrophils were collected and cellular extracts prepared. The supernatants and the cellular extracts were mixed and the levels of TNF-α in the mixture were determined by ELISA assays. n = 3 for each condition; Mean ± SD. Results from two independent experiments are shown.

We have shown above that the secreted levels of TNF-α by PAM stimulated uPAR-/- neutrophils are significantly less than those generated by PAM stimulated WT neutrophils. We next asked if the diminished expression of TNF-α by uPAR-/- neutrophils is due to decreased transcriptional activation. To address this issue, WT and uPAR-/- neutrophils were treated with PAM for 0, 2, or 4 hours and the levels of mRNA for TNF-α and IL-1β were determined by realtime PCR. As shown in Figure 3A, the expression of TNF-α and IL-1β mRNA was similar in PAM stimulated WT and uPAR-/- neutrophils. We also determined the mRNA levels of TNF-α, IL-1β, and IL-6 at early time points after PAM stimulation and did not find significant differences in the expression of these cytokines by WT and uPAR-/- neutrophils (Figure 3B). However, it is possible that the significantly lower levels of TNF-α in the supernatants of PAM treated uPAR-/- neutrophils is caused by a deficiency in the process of cytokine secretion. To test this hypothesis, WT and uPAR-/- neutrophils were lysed after PAM stimulation and the total levels of TNF-α in the supernatants and cellular extracts were determined. As shown in Figure 3C, total levels of TNF-α in the supernatants and cellular extracts from PAM treated uPAR-/- neutrophils were less than those from PAM treated WT neutrophils. These data suggest that although the transcription of cytokines was not different in WT and uPAR-/- cells, there is reduced production of pro-inflammatory cytokines in PAM treated uPAR-/- neutrophils as compared to that by PAM treated WT neutrophils.

### uPAR is not involved in NF-κB activation after TLR2 stimulation in neutrophils

**Figure 4:**
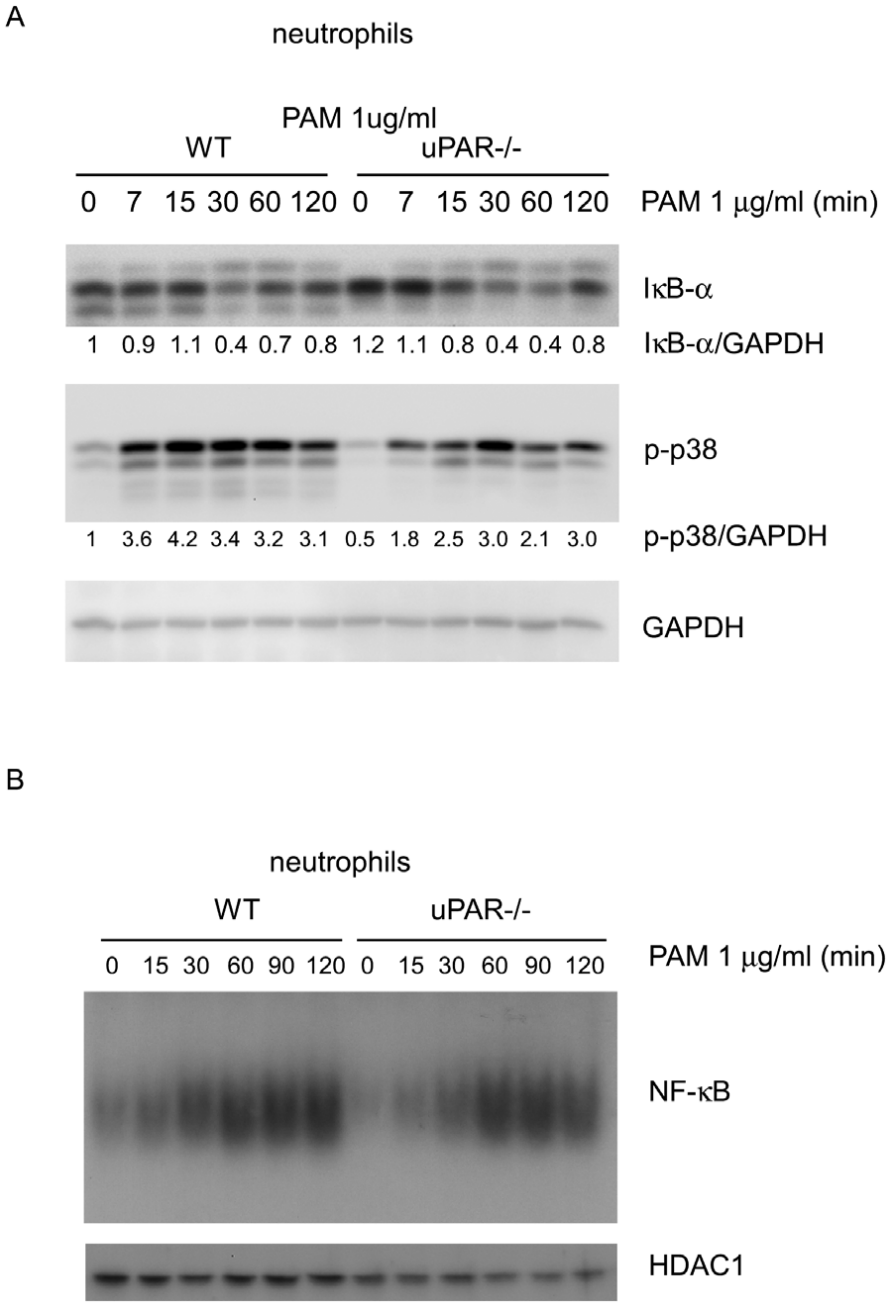
uPAR is not involved in NF-κB activation, but is required for maximal p38 MAPK activation after TLR2 stimulation in neutrophils. (A) 4×10^6^ WT and uPAR-/- neutrophils were treated with 1 µg/ml PAM for 0, 7, 15, 30, 60 or 120 minutes. The cells were collected and cellular extracts prepared. The levels of IκB-α, phosphorylated p38 (p-p38) and GAPDH were determined by Western blotting. The ratios of IκB-α/GAPDH and p-p38/GAPDH were calculated. The ratio in the WT group at time 0 was regarded as 1. The values for all other conditions were the relative ratios to the WT group at time 0. Representative blots from two independent experiments are shown. (B) 4×10^6^ WT and uPAR-/- neutrophils were treated with 1 µg/ml PAM for 0, 15, 30, 60, 90 or 120 minutes. The cells were collected and nuclear extracts prepared. The DNA binding activity of p65 was determined by EMSA assay.

Upon TLR2 stimulation, the TLR2 intracellular domain recruits the adaptor molecule, MyD88, as well as IRAK1 and IRAK4, leading to the activation of kinases, including IKK, p38, and Erk. IKK kinases phosphorylate IκB-α, causing its ubiquitination and degradation by the 26S proteasome and resulting in NF-κB nuclear translocation [1], [2], [3], [4]. To determine if uPAR participates in the regulation of TLR2 related signaling events, we examined the levels of IκB-α in PAM treated WT and uPAR-/- neutrophils. As shown in Figure 4A (IκB-α panel), there was virtually no difference in IκB-α degradation between PAM treated WT and uPAR-/- neutrophils. There was also no difference in DNA binding activity of NF-κB between WT and uPAR-/- neutrophils (Figure 4B). These data, showing similar degrees of degradation of IκB-α and of nuclear translocation of NF-κB in WT and uPAR-/- neutrophils, indicate that uPAR does not contribute in modulating TLR2 induced NF-κB activation.

### uPAR participates in p38 MAPK activation after TLR2 engagement in neutrophils

TLR2 stimulation results in activation of p38 and ERK MAPKs [1], [2], [3], [4]. Since we did not find evidence of involvement of uPAR in regulating TLR2 stimulated IκB-α degradation or NF-κB activation, we next sought to determine if uPAR is involved in MAPK activation after TLR2 stimulation. As shown in Figure 4A (p-p38 panel), phosphorylation of p38 is markedly attenuated in PAM stimulated uPAR-/- as compared to WT neutrophils. However, TLR2 induced ERK activation was comparable between WT and uPAR-/- neutrophils (data not shown). These data suggest that uPAR selectively regulates the TLR2 induced activation of p38, but not Erk.

### TLR2 induced pulmonary inflammatory responses are attenuated in uPAR-/- mice

**Figure 5:**
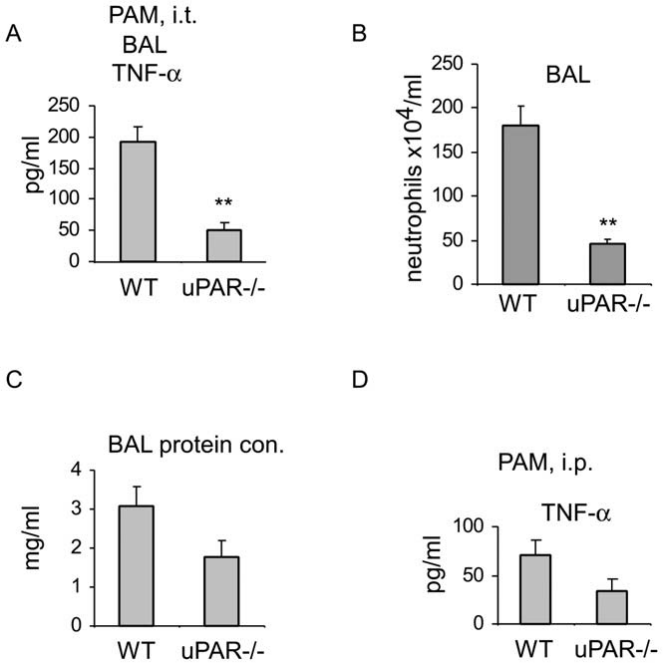
TLR2 induced pulmonary and systemic inflammatory responses are attenuated in uPAR-/- mice. (A–C) WT and uPAR-/- mice were instilled intratracheally (i.t.) with PAM (200 µg/mouse in 50 µl PBS). 24 hours after PAM instillation, the mice were sacrificed and BAL fluids collected. The BAL levels of TNF-α were determined by ELISA (A), neutrophils in the BAL counted (B), and BAL protein concentrations (C) determined. (D) WT and uPAR-/- mice were injected intraperitoneally (i.p.) PAM (200 µg/mouse in 200 µl PBS). 6 hours after PAM injection, blood was withdrawn retro-orbitally and serum prepared. Serum TNF-α levels were determined by ELISA. n = 4–5, mean ± SEM, **p<0.01 when compared to WT group.

In the above experiments, we demonstrated that uPAR participates in modulating TLR2 induced neutrophil activation under *in vitro* conditions. As neutrophils play a major role in mediating acute inflammatory lung injury [26], [27], [28], [29], we next sought to determine if uPAR is involved in regulating inflammatory response and tissue injury in the lungs after TLR2 engagement. As shown in Figure 5A, BAL levels of TNF-α were significantly less in PAM treated uPAR-/- mice than in WT counterparts. Neutrophil numbers in BAL were also significantly lower in PAM treated uPAR-/- as compared to WT mice (Figure 5B). Furthermore, protein concentration in BAL, an index of lung leak, was markedly less in PAM treated uPAR-/- than in WT mice (Figure 5C).

### TLR2 induced systemic inflammatory responses are attenuated in uPAR-/- mice

To define the role of uPAR in the regulation of TLR2 induced inflammatory responses, WT and uPAR-/- mice were given intraperitoneal PAM and serum levels of TNF-α measured 24 hours later. As shown in Figure 5D, serum levels of TNF-α were markedly less in uPAR-/- mice given intraperitoneal PAM than those present in the WT counterparts. These data confirm that uPAR participates in the modulation of TLR2 induced inflammatory responses in vivo.

### uPAR is not required for neutrophil activation upon TLR4 stimulation

**Figure 6:**
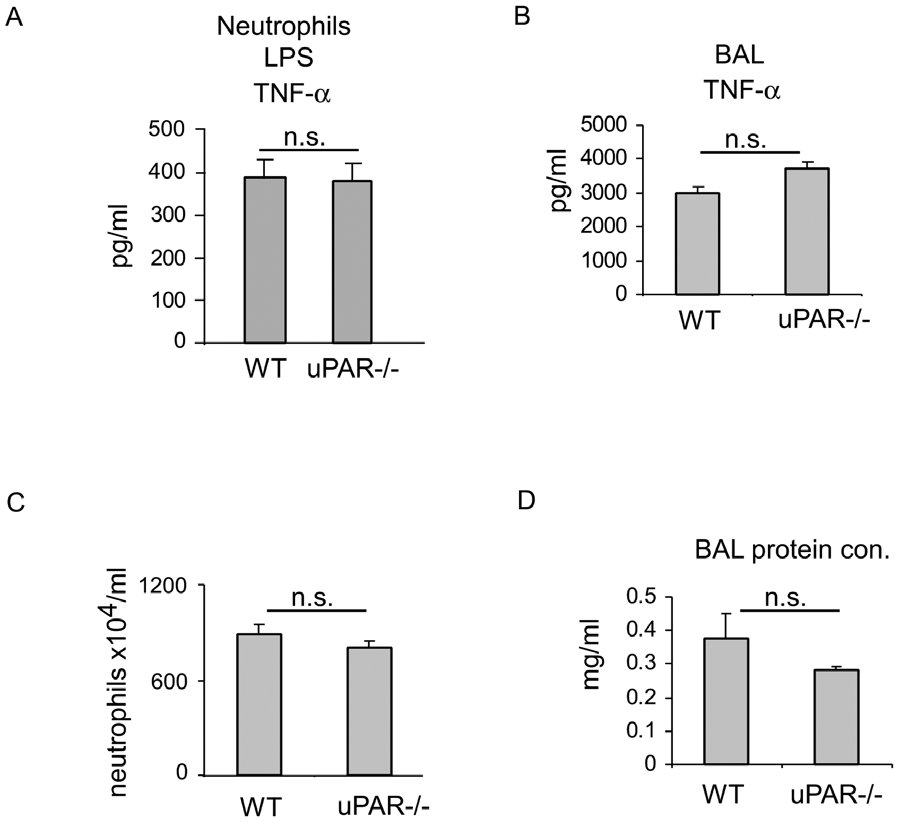
uPAR is not required for neutrophil activation upon TLR4 stimulation. (A) 1×10^6^ WT and uPAR-/- neutrophils were treated with 10 ng/ml LPS for 4 hours and the levels of TNF-α in the supernatants determined. Mean+SD. (B–D) WT and uPAR-/- mice were given LPS intratracheally (50 µg/mouse in 50 µl PBS). 24 hours after LPS instillation, the mice were sacrificed and BAL fluids collected. BAL levels of TNF-α were determined by ELISA (B), neutrophils in BAL counted (C), and BAL protein concentrations determined (D). 4–5 mice in each group, mean ± SEM. All p values were greater than 0.05. n.s. indicates non-significant between groups.

Given the participation of uPAR in TLR2 induced activation of neutrophils, we next asked if uPAR is also required for neutrophil activation upon TLR4 engagement. In these experiments, WT and uPAR-/- neutrophils were cultured with the TLR4 ligand, LPS, and the secreted levels of TNF-α measured. As shown in Figure 6A, there was no difference in the production of TNF-α between LPS treated WT and uPAR-/- neutrophils. These data suggest the uPAR specifically regulates neutrophil activation by TLR2, but not TLR4, stimulation.

### uPAR does not participate in TLR4 induced pulmonary or systemic inflammatory responses

Although our *in vitro* experiments did not demonstrate a role for uPAR in modulating TLR4 induced neutrophil activation, we wished to confirm this lack of interaction between TLR4 and uPAR under *in vivo* conditions. For this purpose, WT and uPAR-/- mice were given LPS intratracheally and inflammatory responses in the lungs determined 24 hours later. As shown in Figures 6B–D, BAL levels of TNF-α, airway neutrophil numbers, and BAL protein concentrations were similar in LPS treated WT and uPAR-/- mice. These data confirm that uPAR is not involved in TLR4 induced neutrophil activation.

### uPAR does not participate in TLR2 or TLR4 induced macrophage activation

**Figure 7:**
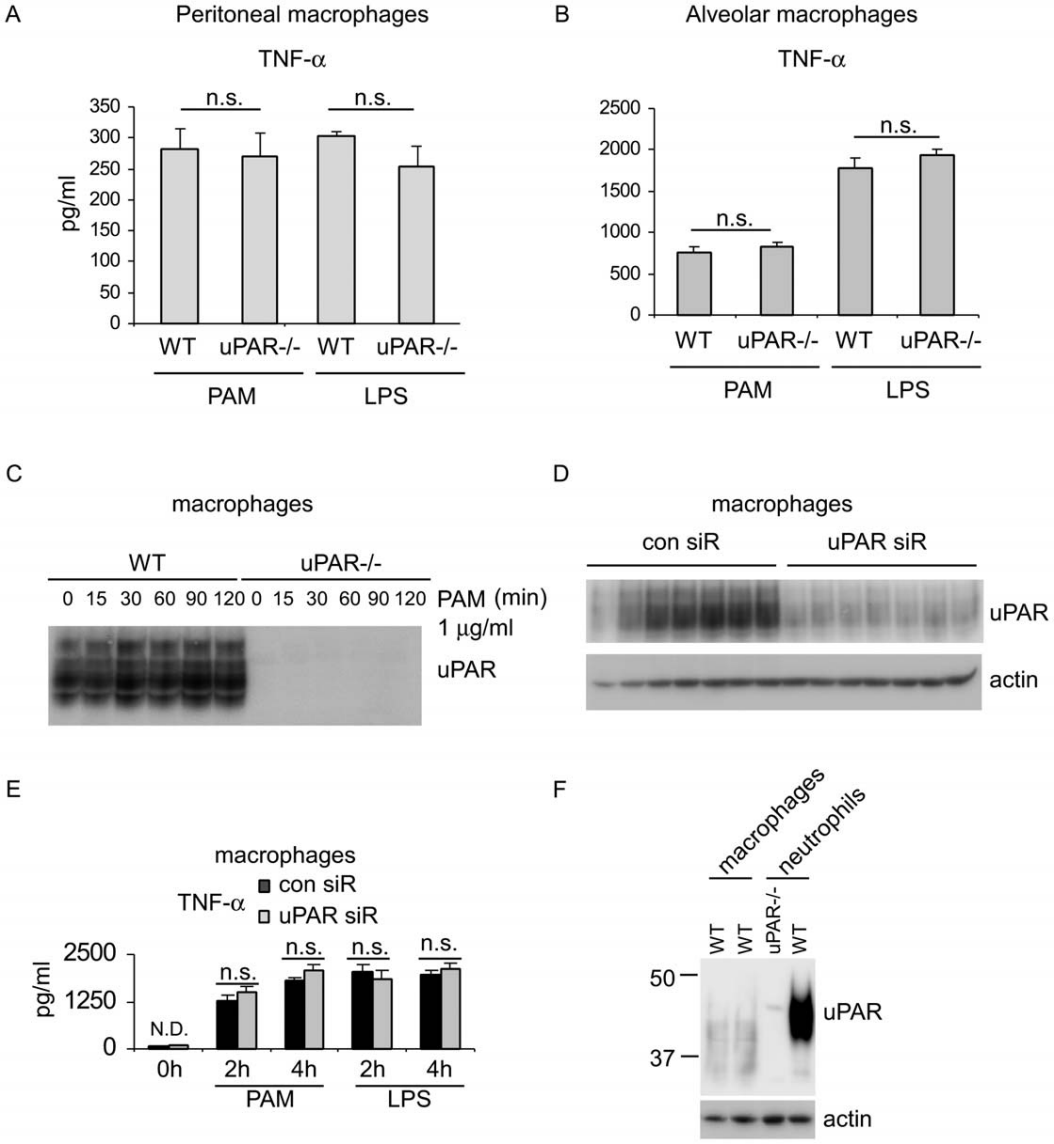
uPAR does not participate in TLR2 or TLR4 induced macrophage activation. (A) 0.25×10^6^ WT and uPAR-/- peritoneal macrophages were treated with 1 µg/ml PAM or 10 ng/ml LPS for 4 hours and the levels of TNF-α in the supernatants determined. (A) 0.2×10^6^ WT and uPAR-/- alveolar macrophages were treated with 1 µg/ml PAM or 10 ng/ml LPS for 4 hours and the levels of TNF-α in the supernatants determined. (C) 1×10^6^ WT and uPAR-/- macrophages were treated with 1 µg/ml PAM for 0, 15, 30, 60 or 120 minutes. The cells were collected and cellular extracts prepared. The levels of uPAR were determined by Western blotting. (D) 1×10^6^ WT and uPAR-/- macrophages were transfected with control siRNA or uPAR siRNA. Three days after transfection, the cells were collected and the levels of uPAR and actin determined by Western blotting. (E) 1×10^6^ WT and uPAR-/- neutrophils were transfected with control or uPAR siRNA. Three days after transfection, the cells were treated with 1 µg/ml PAM or 10 ng/ml LPS for 0, 2, or 4 hours and TNF-α levels in the supernatants measured. (F) The levels of uPAR and actin in WT and uPAR-/- neutrophils and WT macrophages were determined by Western blotting. Results in A, B, C and F were representative of three independent experiments. Mean ± SD; All p values were greater than 0.05. n.s. indicates statistically non-significant differences between groups.

The above experiments demonstrated that uPAR participates in TLR2, but not TLR4 mediated neutrophil activation. To determine if uPAR has similar effects on macrophage activation, peritoneal macrophages from WT and uPAR-/- mice were treated with PAM or LPS. As shown in Figure 7A, the secreted levels of TNF-α were comparable between WT and uPAR-/- peritoneal macrophages after PAM or LPS stimulation. We also performed similar experiments using alveolar macrophages isolated from WT and uPAR-/- mice and found there was no difference in the secreted levels of TNF-α by the alveolar macrophages obtained from the two groups of mice (Figure 7B). Of note, we confirmed that uPAR-/- macrophages demonstrated no uPAR expression (Figure 7C). These data suggest that uPAR does not participate in TLR2 or TLR4 mediated macrophage activation.

To confirm the lack of participation of uPAR in TLR2 or TLR4 induced macrophage activation, we knocked down uPAR in WT macrophages using specific siRNA targeting uPAR. As shown in Figure 7D, uPAR expression was markedly decreased in WT macrophages transfected with uPAR siRNA as compared to those transfected with non-targeting control siRNA. Similar to uPAR-/- macrophages, WT macrophages with uPAR knockdown and stimulated with either LPS or PAM produced TNF-α at levels comparable to WT macrophages transfected with control siRNA (Figure 7E).

To explore mechanisms that might explain the apparently different contribution of uPAR to TLR2 activation in neutrophils and macrophages, we examined the relative expression of uPAR in neutrophils and macrophages. Surprisingly, we found that uPAR from macrophages demonstrated a smaller molecular weight than does uPAR from neutrophils (Figure 7F).

## Discussion

uPAR is a GPI anchored protein that lacks transmembrane and intracellular domains. The primary function of uPAR is to serve as the receptor for uPA, thus concentrating the protease activity of uPA in the proximity of cell surface. uPAR also has protease-independent activities in that it participates in the regulation of cell migration, chemotaxis, cell-cell interaction, and phagocytosis of apoptotic cells [8], [9], [10], [11], [12], [13], [14]. In the present study, we describe a novel role for uPAR in modulating TLR2 mediated neutrophil activation. Consistent with the central role that activated neutrophils play in the development of acute lung injury, we also found that uPAR-/- mice are protected from TLR2 mediated inflammatory response and lung injury.

In these experiments, we found that the presence of uPAR results in enhanced production of the pro-inflammatory cytokines by neutrophils stimulated with PAM, a specific TLR2 ligand. The differential responses to PAM stimulation of WT and uPAR-/- neutrophils were significant in each independent experiment, although statistical analyses were not performed on results combined from multiple independent experiments due to different cell number used each time. However, there was no apparent difference in IκB-α degradation and subsequent NF-κB activation in PAM treated WT and uPAR-/- neutrophils. Consistent with such findings, there was no difference in the transcription of proinflammatory cytokines, such as TNF-α, IL-1β, and IL-6,, as determined by evaluation of mRNA levels, in PAM treated WT and uPAR-/- neutrophils. These data suggest that uPAR is not involved in signaling events downstream of IKK kinase activation in TLR2 stimulated neutrophils, but rather participates in modulating posttranscriptional regulation of the expression of cytokines.

We found no involvement of uPAR in modulating IκB-α degradation in TLR2 activated neutrophils. However, there was markedly less activation of p38 in PAM treated uPAR-/- neutrophils than in PAM treated WT cells. Although TLR stimulation leads to activation of both IKK kinases and MAPKs, previous studies showed that such activation can be divergent in response to some extracellular stimuli [30], [31]. Our data suggest that uPAR may be involved in signaling events leading to activation of MAPKs, specifically p38, in TLR2 stimulated neutrophils. Of note, p38 activation has been shown to regulate post-transcriptional events, such as TNF-α expression, in neutrophils and other cell populations [32], [33], [34], [35]. Defective p38 activation in PAM-treated uPAR-/- neutrophils may contribute to the reduced production of pro-inflammatory cytokines after TLR2 ligation. We also explored potential direct association between uPAR and TLR2. However, we were unable to find any interaction between uPAR and TLR2 or CD14 (data not shown). Therefore, the uPAR associated proteins that differentially regulate PAM induced p38 MAPK activation and neutrophil activation remain to be determined.

We found no apparent involvement of uPAR in TLR4 induced neutrophil activation or in acute lung injury produced by LPS exposure. The mechanisms responsible for the different involvement of uPAR with TLR2 and TLR4 signaling may relate to the distinct requirement for co-factors in TLR4 associated cellular activation [1], [2], [3], [4].

Unlike the potentiating role of uPAR for TLR2 induced neutrophil activation, there was no evidence that uPAR is involved in macrophage activation after TLR2 engagement. The differing participation of uPAR during TLR2 induced activation of neutrophils and macrophages is surprising as, to our knowledge, there is no cellular molecule that differentially regulates the responses of macrophage and neutrophils to TLR stimulation. However, we found that uPAR on the neutrophil surface demonstrated slower migration than did macrophage associated uPAR during PAGE electrophoresis, consistent with a higher molecular weight for neutrophil uPAR. We sequenced uPAR cDNA from neutrophils and macrophages and found that the cDNAs from the two cell populations were identical (data not shown). These data suggest that uPAR on the neutrophil surface differs from that on the macrophage because of posttranslational modifications. These findings are consistent with previous studies in various cell populations demonstrating the presence of different forms of uPAR [25]. Our data suggest that a neutrophil specific modification of uPAR contributes to the regulation of TLR2 mediated activation. Future work will be necessary to further characterize the nature of such neutrophil associated modifications in uPAR and also how such a modification regulates the transduction of TLR2 mediated signaling events.

We found that uPAR-/- mice are protected from TLR2 mediated inflammation and lung injury. Neutrophils play a central role in mediating inflammation in response to infection and tissue injury [26], [27], [28], [29]. Because uPAR-/- neutrophils produce diminished amounts of pro-inflammatory cytokines upon TLR2 stimulation *in vitro*, it is likely that diminished activation of neutrophils *in vivo* contributes to the attenuated inflammatory response and lung injury in PAM treated mice. Previous studies suggested that uPAR plays a detrimental role in hyperoxia-induced lung injury and that uPAR deficiency is associated with diminished neutrophil influx into both lung tissues and bronchoalveolar spaces, accompanied by decreased pulmonary injury [36]. Given that hyperoxia-induced stress was shown to activate TLRs [37], it is of interest to determine if uPAR also regulates hyperoxia induced inflammatory cell activation. Previous studies demonstrated that uPAR expression is upregulated in monocytes by endotoxin [15], [16], [17], [18], [19]. In our study, we did not find a noticeable increase in uPAR expression up to two hours after PAM stimulation in neutrophils. However, it remains possible that uPAR expression may be increased at later time points after PAM treatment and such enhanced expression of uPAR may then contribute to enhanced TLR2 induced neutrophil activation.

Excessive and uncontrolled inflammation after bacterial infection is a leading factor contributing to sepsis, and associated multiple organ dysfunction and death [38], [39]. TLR2 ligands include major components on the surface of both gram-positive and gram-negative bacteria, including peptidoglycans and lipoproteins [1], [2], [3], [4]. Therefore, neutrophil associated uPAR could be a potential target for treating acute inflammation, sepsis, and organ injury related to severe bacterial and other microbial infections in which TLR2 engagement plays a major role.

## Materials and Methods

### Materials

Custom cocktail antibodies and negative selection columns for neutrophil isolation were purchased from StemCell Technologies. Brewer thioglycollate was from Sigma-Aldrich. LPS from *Escherichia coli* 0111:B4 and rabbit anti-actin antibodies were from Sigma-Aldrich. PAM3CSK4 (PAM) was from Invivogene. Antibodies specific for phosphorylated ERK, phosphorylated-p38, total p38, and IκB-α were from Cell Signaling. Goat anti-uPAR was from R&D. Rabbit anti-GAPDH antibodies were from Santa Cruz. Control siRNA and uPAR siRNA were purchased from Dharmacon.

### Mice

uPAR knockout mice that have been backcrossed with C57/BL6 mice for at least nine generations were obtained from Dr. Margaret Gyetko (University of Michigan). Age-and sex-matched control C57/BL6 mice were purchased from NCI-Fredrick. 8-10 week old animals were used for experiments. Animal protocols were approved by the Institutional Animal Care and Use Committee of the University of Alabama at Birmingham.

### Isolation and culture of bone marrow neutrophils

Isolation of bone marrow neutrophils was performed as previously described [40].

### In vivo acute lung injury model (ALI)

The murine ALI model was used as previously described [20], [23]. Briefly, mice were anesthetized with isoflurane. The tongue was then gently extended and a solution of PAM (200 µg PAM/mouse in 50 µl LPS) or LPS (50 µg LPS/mouse in 50 µl PBS) was deposited into the oropharyx. At 24 hours after the PAM or LPS injection, mice were sacrificed and bronchoalveolar lavage (BAL) samples obtained.

### Harvest of bronchoalveolar lavage (BAL) fluid

BAL samples were obtained from PAM or LPS-treated mice by securing a polyethylene catheter in the trachea and then lavaging the lungs three times with 1 ml of iced PBS. Levels of TNF-α in the BAL were determined by ELISA. Total cell counts were measured in the BAL fluid with a hemocytometer and protein concentrations were determined with a Bio-Rad protein assay kit.

### Intraperitoneal injection of PAM in mice

Solutions of PAM (200 µg PAM/mouse in 200 µl PBS) were injected intraperitoneally. At 6 hours after the injections, mice were sacrificed and blood was collected from the retro-orbital venous plexus.

### Western blotting assay

Western blotting assays were performed as previously described [41].

### Cytokine ELISA and protein assays

Immunoreactive TNF-α was quantified using DuoSet ELISA Development kits (R&D Systems) according to the manufacturer's instructions.

### EMSA

Nuclear extracts were prepared and assayed by EMSA as previously described [23]. For analysis of NF-κB, the κB DNA sequence of the Ig gene was used. Synthetic double-stranded sequences (with enhancer motifs underlined) were filled in and labeled with [γ-^32^P] dATP (Perkin-Elmer) using T_4_ polynucleotide kinase as follows: κB sequence, 5′-GCCATGGGGGGATCCCCGAAGTCC-3′ (promega).

### siRNA transfection

WT macrophages were plated in 24 or 48 well plates. One day after cell plating, the cells were transfected with 40 nM control siRNA or uPAR siRNA using HiperFect transfection reagents (Qiagen) according to the manufacturer's instructions. Three days after transfection, the cells were collected for Western blotting or treated with PAM or LPS.

### Statistical analysis

For each experiment, macrophages or neutrophils were isolated and pooled from groups of mice (*n* = 3–4). One representative experiment is shown for most conditions. Each experiments were performed 2–3 times independently, using cells from different groups of mice on different days and showed similar results. One way analysis of variance (ANOVA) for multiple groups followed by the Tukey-Kramer test, or Student's *t* test for comparisons between two groups was used. *p*<0.05 was considered to be statistically significant.
